# Nonlinear association between depressive symptoms and homeostasis model assessment of insulin resistance: a cross-sectional analysis in the American population

**DOI:** 10.3389/fpsyt.2025.1393782

**Published:** 2025-01-22

**Authors:** Chunqi Jiang, Bo Wang, Yinuo Qu, Jun Wang, Xin Zhang

**Affiliations:** ^1^ Treatment of Disease Department, Affiliated Hospital of Shandong University of Traditional Chinese Medicine, Jinan, Shandong, China; ^2^ Department of Pediatrics, Central Hospital of Jinan City, Jinan, Shandong, China; ^3^ College of Acupuncture and Massage, Shandong University of Traditional Chinese Medicine, Jinan, Shandong, China

**Keywords:** non-linear, cross-sectional study, NHANES, depressive symptom, homeostasis model assessment of insulin resistance

## Abstract

**Background:**

Depressive symptom, a pervasive mental health disorder, has garnered increasing attention due to its intricate interconnections with various physiological processes. One emerging avenue of investigation delves into the potential association between depressive symptom and Homeostasis Model Assessment of Insulin Resistance (HOMA-IR), a parameter reflecting insulin resistance. The intricate interplay between these two domains holds promising implications for understanding the multifaceted nature of depressive symptom and its impact on metabolic health.

**Methods:**

We used weighted multivariable logistic regression models with subgroup analysis to explore the relationship between depressive symptom and homeostasis model assessment of insulin resistance. Non-linear correlations were explored using fitted smoothing curves. Then, we constructed a two-piece linear regression model and performed a recursive algorithm to calculate the inflection point.

**Results:**

The study included 20,282 participants in the United States. In the regression model adjusted for all confounding variables, the odds ratio (OR) for the correlation between depressive symptom and the Homeostasis Model Assessment of Insulin Resistance (HOMA-IR) was 1.01 (95% CI: 1.00, 1.01). However, a significant discrepancy between trend tests and regression analyses suggests a potential non-linear relationship between depressive symptom and the assessment of insulin resistance using the Homeostasis Model. Constrained cubic spline analysis confirmed this non-linear relationship, identifying an inflection point at 10.47. Before the inflection point, depressive symptom exhibited a significantly positive correlation with the assessment of insulin resistance using the Homeostasis Model. However, after the inflection point, a negative correlation was observed, though it did not reach statistical significance.

**Conclusion:**

We found a curve-like relationship between depressive symptom and homeostasis model assessment of insulin resistance.

## 1 Introduction

Depression is a prevalent and debilitating mental health disorder that significantly affects individuals’ quality of life worldwide. As per recent studies, it is estimated that Depression impacts over 300 million people globally, representing a substantial burden on both individual and public health systems (1). The adverse effects of Depression extend beyond psychological distress, contributing to considerable physical health risks, including a heightened likelihood of chronic illnesses such as cardiovascular disease and diabetes, thereby amplifying the overall disease burden (2, 3).

The pathogenesis of Depression involves a complex interplay of genetic, neurobiological, and environmental factors. Recent advances in genetic studies have identified specific loci associated with Depression, offering insights into its biological underpinnings (4). Concurrently, environmental factors, such as exposure to stress and adverse life events, have been closely linked to the development of Depression, highlighting the multifaceted nature of this condition (5, 6).

The Homeostatic Model Assessment of Insulin Resistance (HOMA-IR) is a widely utilized index for assessing insulin resistance (IR), a key component in the pathophysiology of metabolic syndrome and type 2 diabetes mellitus. By integrating fasting plasma glucose and insulin levels, HOMA-IR offers a practical and efficient approach for evaluating IR in both clinical and research settings, facilitating early detection and intervention strategies for metabolic disorders (7).

In recent years, studies exploring the link between mood disorders and insulin resistance have been on the rise. Although there is a dearth of studies examining the relationship between depressive symptoms and insulin resistance, opinions among scholars vary. For example, research by Webb M et al. suggests that depressive symptoms are associated with elevated HOMA-IR levels (8). Conversely, Wu CY et al.’s study found no significant overall association between depressive symptoms and insulin resistance (9). Our objective is to leverage the NHANES database, which is both extensive and reliable, to enrich and further investigate the association between depressive symptoms and HOMA-IR.

## 2 Materials and methods

### 2.1 Study participants

**Figure 1:**
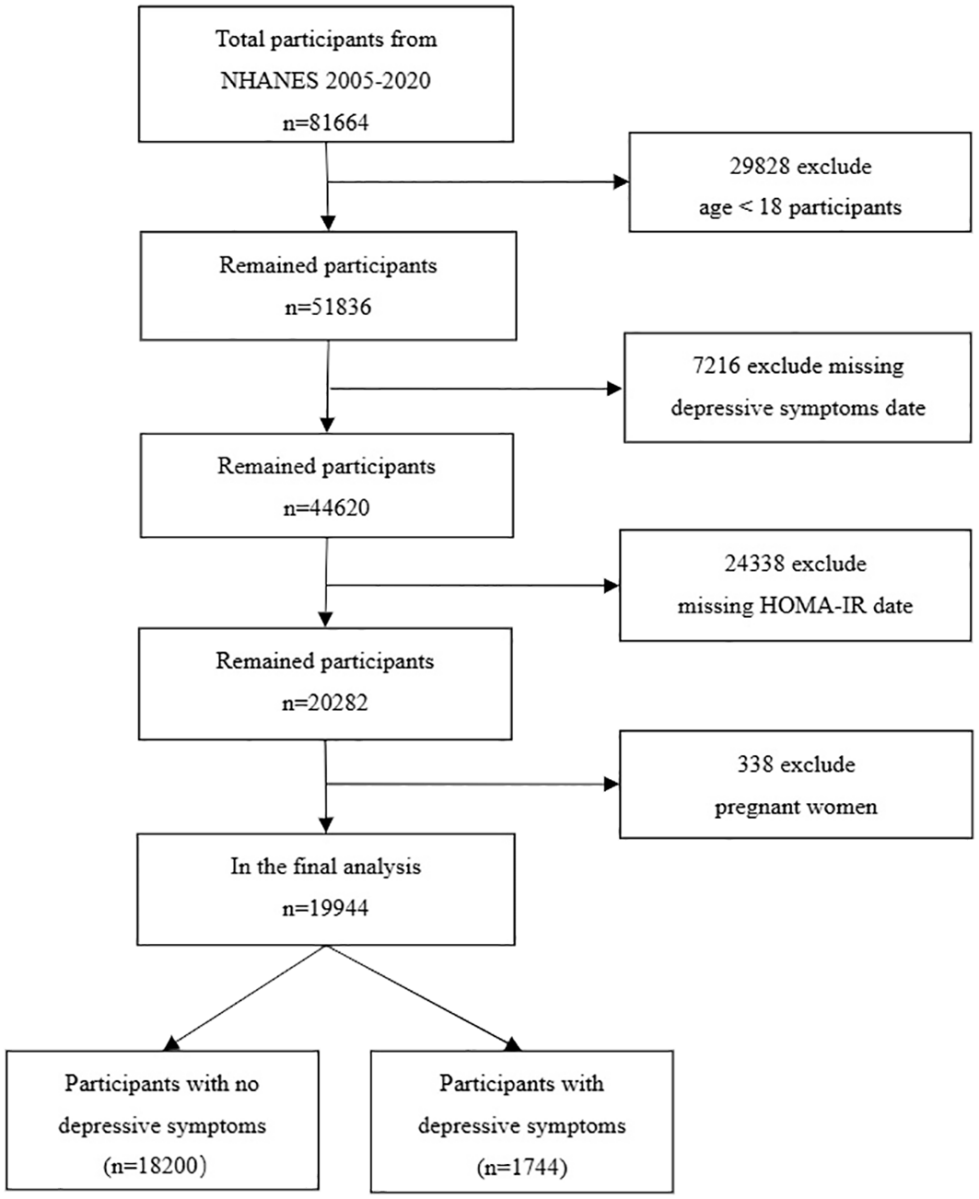
Flow chart of sample selection from the 2005-2020.

Our data was sourced from the United States’ NHANES database. We analyzed data from eight cycles between 2005 and 2020, which provided all the comprehensive information required for our study. NHANES, conducted by the National Center for Health Statistics, is a cross-sectional survey that includes a nationally representative sample of the non-institutionalized civilian U.S. population. Executed biennially, NHANES employs a stratified, multistage probability sampling approach and covers demographic, socioeconomic, health-related, and medical information. The NHANES research protocol is approved by the NCHS Research Ethics Review Board, and no additional ethical review was needed for this study. By pooling data from these eight cycles, we amassed a total of 49,461 participants. Our study’s participants were filtered based on the following exclusion criteria: 1. Age under 18; 2. Incomplete Patient Health Questionnaire-9 (PHQ-9); 3. Absence of HOMA-IR data; 4. Pregnant women. Following the exclusion of 29,828 participants under 18 years of age, 7,216 subjects lacking data on depressive symptoms, 24,338 subjects without HOMA-IR information, and 338 pregnant women, we were left with a study cohort of 19,944 subjects (**Figure 1**).

### 2.2 Study variables

#### 2.2.1 *Definition* of depressive symptom

The survey utilized the 9-Item Patient Health Questionnaire (PHQ-9) to evaluate symptoms of depression that occurred within the two weeks leading up to the assessment. Designed to align with the Diagnostic and Statistical Manual of Mental Disorders, Fourth Edition, the PHQ-9 targets the nine specific diagnostic indicators of major depressive disorder, which include feelings of sadness, diminished interest in activities, sleep irregularities, tiredness, reduced self-worth, changes in appetite, concentration difficulties, noticeable restlessness or slowed behavior, and thoughts of self-harm. Scores for each question were defined as 0 (none), 1 (a few days), 2 (more than half the days), or 3 (almost every day), and total scale scores ranged from 0 to 27. A person with a total score of 10 on the PHQ-9 was considered to have major depressive disorder; the sensitivity of this cut-off value was 88%, and the specificity was 88% (10).

#### 2.2.2 Definition of HOMA-IR

A blood sample was obtained from participants who underwent a morning physical examination after fasting for at least 8 hours. The plasma glucose level was determined using the COBRAS MIRA hexokinase enzymatic reference method (Roche Diagnostics, Indianapolis, IN), while the serum insulin level was measured using a radio-immunoassay (Pharmacia Diagnostics, Uppsala, Sweden). To assess insulin resistance (IR), which is commonly evaluated in large epidemiological studies, we utilized the homeostasis model assessment of IR (HOMA-IR) formula: fasting serum insulin (μU/mL) multiplied by fasting plasma glucose (mmol/L), divided by 22.5 (7).

#### 2.2.3 Assessment of other variables

The CDC collected demographic, lifestyle, self-reported health, physical measurement, and biochemical data systematically and comprehensively, in the form of individual interviews with computer assistance. In our study, the main demographic variables required were the participants’ age, sex, ethnicity, education, and income poverty rate; lifestyle variables we considered included the participants’ smoking status, alcohol consumption, and recreational activities; physical variables included BMI; health status: diabetes, hypertension, hyperlipidemia, heart disease, stroke. Smoking status: Never: participants had smoked less than 100 cigarettes so far; Former: participants had a history of smoking but did not currently smoke; Now: participants were still smoking (11). Recreational activities: “yes” and “no”. Stroke was determined by the question MCQ160f, which specifically asked, “Have you ever been told by a doctor or other health professional that you have had a stroke?” Cardiovascular disease was obtained from the Medical Status Questionnaire, which recorded whether participants had been informed by a doctor that they had coronary heart disease, congestive heart failure, or a heart attack (12). Diabetes diagnosis (diabetes and prediabetes): at least one of the following: 1. Fasting plasma glucose (FPG >7.0 mmol/L). 2. Glycosylated hemoglobin HbA1c (>= 6.5%). 3. Random plasma glucose (>= 11.1mmol/L). 4. 2 h OGTT plasma glucose (>= 11.1mmol/L). 5. The doctor says you have diabetes. 6. IFG (6.11mmol/L <=FPG <=7.0mmol/L). 7. IGT (7.7mmol/L <=OGTT <=11.1mmol/L). Hypertension: At least one of the following. 1. Systolic blood pressure ≥140mmhg. 2. Diastolic blood pressure ≥90mmhg. 3. Taking antihypertensive medication. 4. Self-reported hypertension. Hyperlipidemia: One of the following. 1. Triglycerides ≥150mg/dL. 2. Total cholesterol ≥200mg/dL. 3. Low-density lipoprotein (LDL) ≥130mg/dL. 4. High-density lipoprotein (HDL): ≤50mg/dL for women, ≤40mg/dL for men. 5. Taking lipid-lowering medication. Drinking status: Heavy (women: ≥3 drinks per day or ≥4 drinks in one sitting); Men: ≥4 drinks per day or ≥5 drinks in one sitting; binge drinking ≥5 days per month); Moderate (women: ≥2 drinks per day; Men: ≥3 drinks per day; binge drinking ≥2 days per month); Mild (women: 1 drink per day; Men: 2 drinks per day); Former (drank before, but no longer); Never (less than 12 drinks in a lifetime) (13).

### 2.3 Statistical analysis

The data analysis was conducted using weights in accordance with NCHS guidelines to ensure the sample’s representativeness. Missing variables were subjected to imputation. Missing data were imputed using predictive mean matching for numeric variables and logistic regression for binary variable. Participants were categorized into two groups based on the presence or absence of depressive symptom to assess baseline characteristics. Mean (95% CIs) depicted continuous variables, while percentages described categorical variables. To compare the baseline characteristics of continuous and categorical variables, we employed weighted linear regression for continuous variables, chi-square tests for unordered categorical variables, and Mann-Whitney U tests for ordered categorical variables. We conducted a weighted multiple logistic regression analysis to explore the association between HOMA-IR and depressive symptoms. The findings were presented through odds ratios (ORs) with 95% confidence intervals (95% CI). HOMA-IR was categorized into four equal quartiles, designated as Q1, Q2, Q3, and Q4, to assess the stability of the relationship under examination. To examine consistency and variations in the association across different population subgroups, stratified analyses along with linear trend tests were performed. The existence of a non-linear relationship between depressive symptom and HOMA-IR was evaluated using restricted cubic splines, and a recursive algorithm along with a two-piece linear regression model was employed to identify the inflection point in cases of nonlinearity. R software (version 4.2.0) and Empower Stats were utilized for statistical analyses, with statistical significance set at p < 0.05.

## 3 Results

### 3.1 Baseline characteristics

**Table 1** shows the baseline characteristics of the participants. As can be seen from **Table 1**, in patients with depressive symptom, they have a larger BMI, HOMA-IR. Participants without depressive symptoms had an average age of 47.15 (46.63, 47.68), while those with depressive symptoms had an average age of 47.14 (46.20, 48.09). The prevalence of depressive symptom is higher among people with low education, women, hypertension, hyperlipidemia, stroke, cardiovascular and cerebrovascular diseases, and non-recreational activities.

**Table 1:** Baseline characteristics of participants.

	No depressive symptoms (n=18200)	With depressive symptoms (n=1744)	P-value
Age (year)	47.15 (46.63,47.68)	47.14 (46.20,48.09)	0.9860
Sex (%)			<0.0001
Female	48.96 (48.07,49.84)	63.38 (60.38,66.28)	
Male	51.04 (50.16,51.93)	36.62 (33.72,39.62)	
Race/ethnicity (%)			<0.0001
Mexican American	8.66 (7.55,9.93)	7.89 (6.29,9.84)	
Non-Hispanic White	68.29 (66.00,70.48)	63.36 (59.68,66.90)	
Non-Hispanic Black	10.15 (9.01,11.41)	13.69 (11.71,15.94)	
Other Hispanic	5.49 (4.72,6.37)	8.05 (6.38,10.10)	
Other Race	7.42 (6.70,8.20)	7.01 (5.62,8.72)	
Marry status (%)			<0.0001
Never married	17.51 (16.35,18.73)	20.35 (17.74,23.23)	
Married/Living with partner	65.03 (63.62,66.42)	48.43 (44.77,52.12)	
Divorced/Widowed/Separated	17.46 (16.59,18.38)	31.22 (28.40,34.18)	
Education status (%)			<0.0001
Less than high school	4.58 (4.11,5.09)	8.83 (7.53,10.33)	
High school	34.39 (32.82,35.99)	43.51 (40.25,46.82)	
More than high school	61.04 (59.27,62.77)	47.67 (44.05,51.31)	
Recreational activity (%)			<0.0001
No	44.25 (42.62,45.90)	65.23 (61.35,68.92)	
Yes	55.75 (54.10,57.38)	34.77 (31.08,38.65)	
Drinking status (%)			<0.0001
Never	10.66 (9.82,11.55)	10.50 (9.15,12.02)	
Mild	38.97 (37.60,40.35)	27.15 (23.91,30.64)	
Moderate	17.90 (17.08,18.76)	17.47 (14.67,20.68)	
Heavy	20.96 (19.99,21.96)	29.13 (26.16,32.29)	
Former	11.51 (10.70,12.38)	15.75 (13.09,18.84)	
Smoking status (%)			<0.0001
Never	55.85 (54.49,57.20)	38.05 (35.01,41.18)	
Now	18.17 (17.11,19.28)	38.56 (35.24,41.98)	
Former	25.97 (24.89,27.08)	23.40 (20.39,26.69)	
Hypertension (%)			<0.0001
No	63.13 (61.88,64.37)	51.36 (47.72,54.98)	
Yes	36.87 (35.63,38.12)	48.64 (45.02,52.28)	
Cardiovascular Disease (%)			<0.0001
No	91.25 (90.60,91.86)	82.96 (80.53,85.14)	
Yes	8.75 (8.14,9.40)	17.04 (14.86,19.47)	
Hyperlipidemia (%)			<0.0001
No	30.45 (29.35,31.58)	24.40 (21.83,27.18)	
Yes	69.55 (68.42,70.65)	75.60 (72.82,78.17)	
Income to poverty ratio	3.08 (3.02,3.14)	2.19 (2.07,2.32)	<0.0001
Stroke (%)			<0.0001
No	97.21	92.96	
Yes	2.79	7.04	
BMI (kg/m^2^)	28.91 (28.74,29.08)	30.77 (30.33,31.22)	<0.0001
HOMA-IR	3.70 (3.59,3.81)	4.91 (4.57,5.25)	<0.0001

P (Continuous variables): calculated by weighted linear regression model. P (Categorical variables): calculated by weighted chi-square test. HOMA-IR, homeostasis model assessment of insulin resistance; BMI, body mass index.

### 3.2 Association between depressive symptom and HOMA-IR

We used multiple logistic regression equation, sensitivity analysis and trend test to explore the relationship between depressive symptom and HOMA-IR, and the results were shown in **Table 2**. In model 1 (unadjusted model), OR (95%CI) was 1.02 (1.01, 1.02), indicating a significant positive correlation between depressive symptom and HOMA-IR. In model 3, We adjusted for sex, age, race, hyperlipidemia, family income to poverty ratio, education level, smoking status, marriage status, drinking status, physical activity and other covariates, OR (95%CI) is 1.01 (1.00, 1.01). It also showed that there was a significant positive correlation between depressive symptom and HOMA-IR, but the correlation was weaker than model 1. HOMA-IR was stratified into four quartiles, designated as Q1, Q2, Q3, and Q4. In Model 3, taking Q1 as the reference category, the odds ratios (OR) and 95% confidence intervals (CI) for Q2, Q3, and Q4 were 1.07 (0.88, 1.29), 0.97 (0.79, 1.19), and 1.20 (0.96, 1.50), respectively. The p-value for trend across these quartiles exceeded 0.05. This indicates that there may be a curvilinear relationship between depressive symptom and HOMA-IR. In the sex-stratified analysis, there was a significant positive correlation between depressive symptom and HOMA-IR, which was consistent with the results without stratification. When stratified by age and BMI, a significant positive association was found between depressive symptom and HOMA-IR in participants over 60 years old and obese. The association between depressive symptom and HOMA-IR was not significant in participants under 60 years of age and BMI<=30.

**Table 2:** Association of depressive symptom and HOMA-IR.

Exposure	Model 1 OR, (95%CI)	Model 2 OR, (95%CI)	Model 3 OR, (95%CI)
HOMA-IR	1.02 (1.01, 1.02)	1.02 (1.01, 1.02)	1.01 (1.00, 1.01)
HOMA-IR quartile			
Q1	Reference	Reference	Reference
Q2	1.10 (0.95, 1.28)	1.10 (0.95, 1.27)	1.07 (0.88, 1.29)
Q3	1.10 (0.94, 1.27)	1.11 (0.95, 1.28)	0.97 (0.79, 1.19)
Q4	1.80 (1.57, 2.06)	1.82 (1.59, 2.10)	1.20 (0.96, 1.50)
P for trend	<0.0001	<0.0001	0.2176
Stratified by sex			
Female	1.03 (1.02, 1.03)	1.03 (1.02, 1.03)	1.01 (1.00, 1.02)
Male	1.01 (1.01, 1.02)	1.01 (1.01, 1.02)	1.01 (1.00, 1.02)
Stratified by sex			
< = 60	1.02 (1.01, 1.03)	1.02 (1.02, 1.03)	1.00 (1.00, 1.01)
> 60	1.02 (1.01, 1.02)	1.02 (1.01, 1.02)	1.01 (1.00, 1.02)
Stratified by BMI			
<=25	1.03 (1.01, 1.05)	1.03 (1.01, 1.05)	1.00 (0.93, 1.07)
25 - 30	1.01 (1.00, 1.02)	1.01 (1.00, 1.03)	1.00 (0.98, 1.02)
= >30	1.01 (1.01, 1.02)	1.02 (1.01, 1.02)	1.01 (1.00, 1.01)

Model 1: no adjustment.

Model 2: adjusted for age, sex, and race.

Model 3: adjusted for sex, age, BMI, hypertension, hyperlipidemia, family income to poverty ratio, education level, smoking status, marriage status, drinking status, physical activity, stroke, cardiovascular disease and diabetes.

In stratified analyses by age, sex, or BMI, the relationship between HOMA-IR and depressive symptoms was assessed without adjusting for the stratification variables. Abbreviations: HOMA-IR, homeostasis model assessment of insulin resistance; BMI, body mass index; OR, odds ratios; CI, confidence intervals.

**Figure 2:**
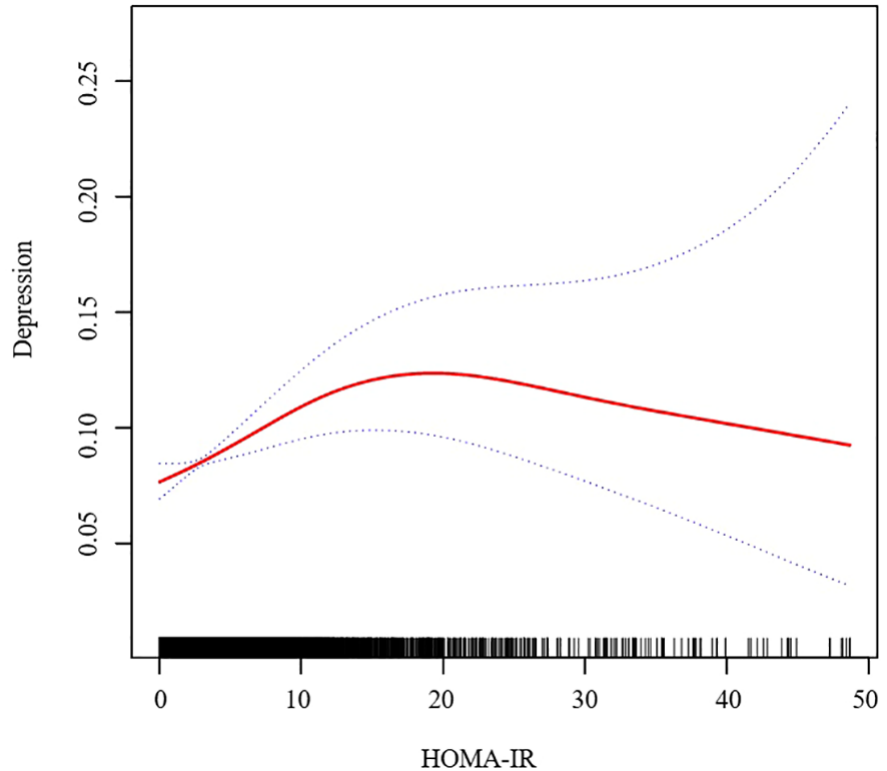
Association between HOMA-IR and Depressive symptom. We adjusted for sex, age, BMI, hypertension, hyperlipidemia, family income to poverty ratio, education level, smoking status, marriage status, drinking status, physical activity, cardiovascular disease and diabetes. HOMA-IR, homeostasis model assessment of insulin resistance; BMI, body mass index.

**Figure 3:**
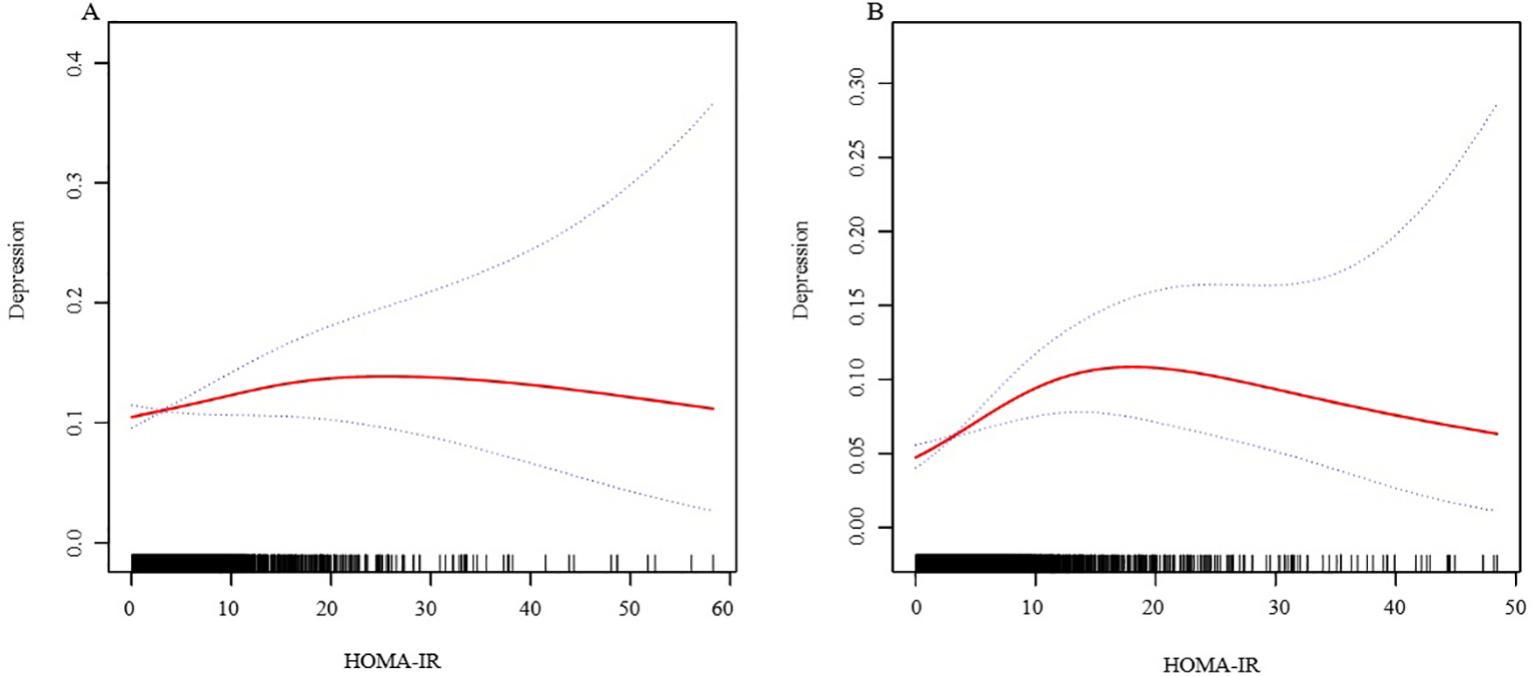
The association between HOMA-IR and depressive symptoms, analyzed with stratification by sex. (**A**: Female; **B**: Male). We adjusted for sex, age, BMI, hypertension, hyperlipidemia, family income to poverty ratio, education level, smoking status, marriage status, drinking status, physical activity, cardiovascular disease and diabetes. HOMA-IR, homeostasis model assessment of insulin resistance; BMI, body mass index.

**Figure 4:**
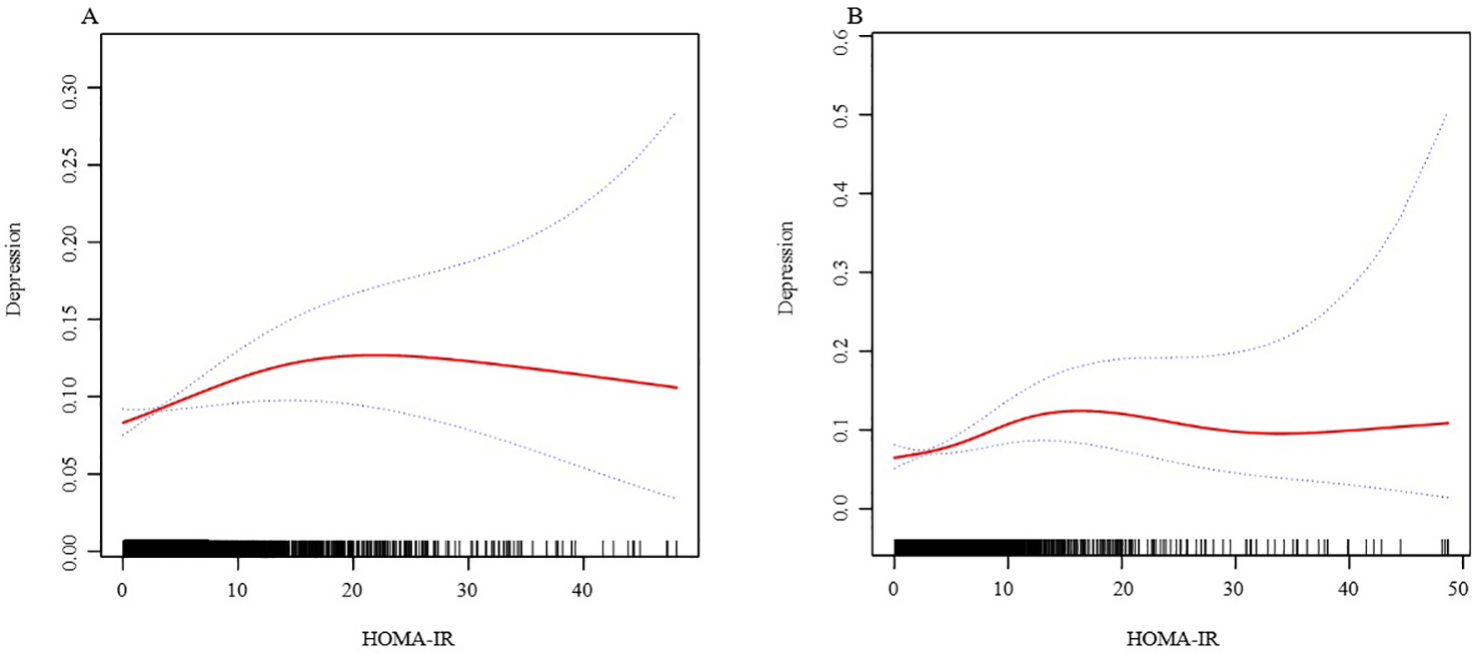
The association between HOMA-IR and depressive symptoms, analyzed with stratification by age. (**A**: age<=60; **B**: age>60). We adjusted for sex, BMI, hypertension, hyperlipidemia, family income to poverty ratio, education level, smoking status, marriage status, drinking status, physical activity, cardiovascular disease and diabetes. HOMA-IR, homeostasis model assessment of insulin resistance; BMI, body mass index.

In further analysis, we used GAM and smoothing curve to analyze the association between Depressive symptom and HOMA-IR. We have established the curvilinear relationship between depressive symptoms and HOMA-IR, as depicted in **Figure 2**, and have determined the inflection point to be at 10.47, as detailed in **Table 3**. Before the inflection point, OR (95%CI) was 1.05 (1.02, 1.08), indicating a significant positive correlation between Depressive symptom and HOMA-IR. After the inflection point, OR (95%CI) was 0.99 (0.96, 1.01), indicating that Depressive symptom was negatively correlated with HOMA-IR, but the correlation was not significant. In subgroup analyses stratified by age or sex, this relationship remains non-linear (**Figures 3**, **4**).

**Table 3:** Threshold effect analysis of HOMA-IR on Depressive symptom using a two-piecewise linear regression model.

Outcome:	Depressive symptom
Fitting by standard linear model	1.01 (1.00, 1.03) 0.1502
Fitting by two-piecewise linear model	
Inflection point	10.47
< 10.47	1.05 (1.02, 1.08) 0.0022
> 10.47	0.99 (0.96, 1.01) 0.2699
Log-likelihood ratio	0.007

We adjusted for sex, age, BMI, hypertension, hyperlipidemia, family income to poverty ratio, education level, smoking status, marriage status, drinking status, physical activity, cardiovascular disease and diabetes.

In subgroup analyses (**Table 4**), we found consistent interactions across all variables, suggesting a positive association between Depressive symptom and HOMA-IR in these subgroup analyses.

**Table 4:** Subgroup analysis.

Subgroup	OR (95%CI)	P	P for interaction
Cardiovascular Disease			0.1182
No	1.00 (0.99, 1.01)	0.4016	
Yes	1.01 (1.00, 1.02)	0.0051	
Diabetes			0.6370
No	1.02 (0.98, 1.06)	0.2534	
Yes	1.01 (1.00, 1.01)	0.0462	
Prediabetes	1.02 (0.98, 1.07)	0.3829	
Smoking status			0.5027
Never	1.01 (1.00, 1.02)	0.1243	
Former	1.01 (1.00, 1.02)	0.0128	
Now	1.00 (0.98, 1.02)	0.9209	
Stroke			0.7973
No	1.01 (1.00, 1.01)	0.0214	
Yes	1.01 (1.00, 1.03)	0.1883	
Drinking status			0.2634
Never	1.02 (1.00, 1.04)	0.0760	
Mild	1.00 (0.99, 1.02)	0.4419	
Moderate	1.02 (0.99, 1.04)	0.1777	
Heavy	1.02 (1.00, 1.04)	0.0177	
Former	1.00 (0.98, 1.02)	0.8766	
Recreational activity			0.3783
No	1.01 (1.00, 1.02)	0.0075	
Yes	1.00 (0.99, 1.02)	0.4664	
Marry status			0.4003
Never married	1.01 (0.99, 1.04)	0.2333	
Married/Living with partner	1.01 (1.00, 1.02)	0.0097	
Divorced/Widowed/Separated	1.00 (0.99, 1.01)	0.9031	

We adjusted for sex, age, BMI, hypertension, hyperlipidemia, family income to poverty ratio, education level, smoking status, marriage status, drinking status, physical activity, stroke, cardiovascular disease and diabetes but not adjusted for the subgroup analysis variables themselves. OR, odds ratios; CI, confidence intervals.

## 4 Discussion

In the research, after adjusting for all potential covariates, we observed a positive association between HOMA-IR and depressive symptom. With the increase in HOMA-IR, the incidence rate of depressive symptom also increased. Furthermore, upon conducting spline curve fitting, we discovered this positive correlation to be nonlinear, with a turning point at 10.47. Before this turning point, for every unit increase in HOMA-IR, the risk of depressive symptom increased by 5%. After the turning point, each unit increase in HOMA-IR was associated with a 1% decrease in depressive symptom risk, although this was not statistically significant. To our knowledge, this is the first large-scale study to explore the relationship between HOMA-IR levels and the odds of depressive symptom.

The relationship between HOMA-IR and depressive symptoms is a subject of debate, with studies presenting divergent findings. Consistent with our results, a study by Diniz BS et al. on Mexican-American elderly individuals discovered an association between depressive symptoms and insulin resistance (14). Similarly, research by He Y et al. demonstrated that the risk of IR escalates with increasing levels of depressive status among obese women (15). However, some studies have contradicted these findings. Geraets AFJ et al.’s research indicated that HOMA-IR is not associated with the risk of developing depressive symptoms (16). Landucci Bonifácio K et al. also arrived at a similar conclusion, stating that mood disorders, including major depression, are not linked to an increased risk of IR (17).

The pathogenesis of depression is complex, integrating genetic, neurobiological, and environmental factors. Recent research underscores the significance of inflammation, neurotransmitter dysfunction, and neuroendocrine disturbances in depression. Inflammation, marked by elevated levels of cytokines such as interleukin-6 (IL-6) and tumor necrosis factor-alpha (TNF-α), has been directly associated with depression, suggesting an inflammatory component to the disorder (18, 19). Neuroendocrine dysregulation, particularly within the hypothalamic-pituitary-adrenal (HPA) axis, manifests in altered cortisol levels, further contributing to depression’s etiology (20, 21). Additionally, imbalances in neurotransmitters such as serotonin and dopamine are pivotal in mood regulation disturbances characteristic of depression (22, 23). Insulin exerts a direct influence on serotonergic and dopaminergic neurotransmission, and IR affects these neurotransmitter systems, thereby impacting depressive symptoms (24). Moreover, IR promotes the accumulation of fatty acids and triglycerides within cells, which triggers inflammation and raises the levels of pro-inflammatory cytokines, including TNF-α and IL-6 (25). Furthermore, IR increases the metabolic demands on individuals with mood disorders, potentially leading to more significant neurofunctional deficits and cognitive impairments, such as difficulties in verbal communication, numerical reasoning, and mental processing speed (26). According to Bruce S McEwen (27), the body may trigger compensatory biological responses when it is in a state of high insulin resistance and inflammation, regulating this adverse condition. This could be the reason for the existence of a saturation threshold between HOMA-IR and depression.

To better explore the underlying truths, we conducted subgroup analyses. In the stratified analysis by sex and age, we found no significant sex differences in the relationship between HOMA-IR and depression. However, an independent association was observed within the obese population. Upon performing stratified smooth curve fitting, we discovered that the relationships across sex and age groups were nonlinear. Furthermore, there was no significant interaction between HOMA-IR and other related risk factors, indicating that no additional factors were found to significantly influence the association between HOMA-IR and depression. Obesity is a significant risk factor for the development of insulin resistance. The excessive adipose tissue in obesity leads to chronic inflammation and the release of pro-inflammatory cytokines, thereby impairing insulin signaling and leading to insulin resistance (28). Concurrently, obesity-related inflammation is also associated with the development of depression. Pro-inflammatory cytokines can cross the blood-brain barrier, affecting brain function and leading to mood disorders (29, 30). Additionally, adipokines released by adipose tissue, such as leptin and adiponectin, play roles in both metabolic regulation and mood regulation. The dysregulation of these adipokines in obesity may contribute to the pathophysiology of both insulin resistance and depression (31, 32).

Our study presents several significant strengths that enhance the robustness and applicability of its conclusions. Firstly, the large volume of data collected has increased the reliability of our findings across diverse populations. Secondly, the strict standards applied during the data collection process ensure high data integrity and accuracy, substantially reducing the risk of biases that could distort the study’s outcomes. Despite these advantages, our research is not without its limitations. The temporal span of the data, while beneficial for capturing a broad range of information, may also introduce variability and systematic errors that could impact the observed associations. Relying solely on the PHQ-9 scale to assess depressive symptoms, our results may be susceptible to biases arising from patient self-reporting, such as misunderstandings or deliberate omissions. The inherent limitations of a cross-sectional study design restrict our ability to establish causal relationships between HOMA-IR levels and depression. Although we can observe associations between them, determining whether increased insulin resistance leads to depression, whether depression contributes to changes in insulin sensitivity, or if a bidirectional relationship exists, remains challenging.

## 5 Conclusion

Our study, analyzing NHANE database data from 2003 to 2020, discovered a non-linear positive correlation between HOMA-IR and depressive symptom in the US adult population, even after controlling for confounders. We identified a saturation threshold, indicating a limit to how insulin resistance impacts depressive symptom severity. This highlights the complex relationship between metabolic health and mental well-being, emphasizing the need for holistic approaches in managing both insulin resistance and depressive symptom. This research contributes to understanding the nuanced interactions between physical and mental health, advocating for integrated treatment strategies.

## Data Availability

The original contributions presented in the study are included in the article/**Supplementary Material**. Further inquiries can be directed to the corresponding author.
